# Significance of heme oxygenase-1(*HMOX1*) gene on fetal hemoglobin induction in sickle cell anemia patients

**DOI:** 10.1038/s41598-020-75555-y

**Published:** 2020-10-28

**Authors:** Priya Hariharan, Vrushali Chavan, Anita Nadkarni

**Affiliations:** Department of Haematogenetics, ICMR-National Institute of Immunohaematology, 13th floor NMS Building, KEM Hospital Campus, Parel, Mumbai, 400012 Maharashtra India

**Keywords:** Genetics, Molecular biology, Biomarkers

## Abstract

Though the patients with sickle cell anemia (SCA) inherit same genetic mutation, they show considerable phenotypic heterogeneity. It has been observed that patients with elevated fetal hemoglobin (HbF) levels have a relatively mild clinical course. There is sparse literature on the association of higher HbF levels leading to reduction in the oxidative stress in SCA patients. Hence in this study, the significance between the *HMOX1* gene polymorphisms and the HbF levels has been studied. Preliminary screening was carried out. Genotyping of 3 variants in the *HMOX1* gene was performed in 90 SCA patients and 50 healthy controls by PCR–RFLP, GeneScan and direct DNA sequencing. It was observed that SCA patients with higher HbF levels, showed improved hematological indices with an inverse effect on HbS levels. The TT genotype of rs2071746 (A→T) polymorphism was found to be associated with elevated HbF levels (P: 0.012). Also, the long form (> 25 GT repeats) of rs3074372 (GT)n repeats was found to be linked with increased HbF levels. We could not find any association of rs2071749 (A→G) polymorphism with the HbF levels. As, the sickle cell anemia patients show significant oxidative stress due to hemolysis, the study of polymorphisms in the *HMOX1* gene may act as a potential independent marker for elevated HbF levels.

## Introduction

Sickle cell anemia (SCA) is an inherited blood disorder which is caused due to mutation in the beta globin gene that results in the substitution of Glutamic acid with Valine at 6th amino acid position of the β-globin chain. The resultant variant hemoglobin, under deoxygenic phase tends to polymerise in the red blood cells, leading to an altered erythrocyte shape with increased rigidity and rheological properties. This is further followed with a cascade of painful events, that lead to episodes of vasocclusive crisis and ischemia reperfusion injury^1^. The mechanism that leads to oxidative burden in sickle cell patients is excess levels of free heme in the plasma that auto‐oxidizes inducing methemoglobin and superoxide formation^2^. The free heme due to its hydrophobicity easily perturbs the cell membrane, thus increasing its susceptibility to generate reactive oxygen species (ROS). The exemplified ROS production, oxidises lipids, proteins and damages DNA by further triggering cell-signalling pathways that induce apoptosis and cell death. ROS also alters the hemorheological properties in SCA patients by increased oxidation of the RBC membrane bound proteins^3,4^. The additive effect of these clinical conditions generated by excessive ROS, aggravate the symptoms in SCA. Heme oxygenase (HO) enzyme encoded by *HMOX1* gene, located in the long arm of chromosome 22, plays a central role in catabolism of the toxic free heme to 3 biologically active by products i.e. carbon monoxide (CO), iron and biliverdin. The enzyme occurs as 2 isozymes, an inducible heme oxygenase-1 (HO-1) which is induced in presence of free heme and a constitutive heme oxygenase-2^5^. The by-products of heme metabolism due to the HO-1, act as potent scavengers of ROS and display an anti-inflammatory effect. It has also been shown that, murine cells devoid of HMOX1 gene expression, were more susceptible to oxidative injury^6^. Thus HO-1 acts as a potential cytoprotective and anti-inflammatory enzyme which is activated at times of inflammation, hypoxia and ischemic condition. HMOX-1 also plays an important role in healing and in re-establishment of hemostasis^7^.

It is a well-established fact that elevated HbF levels reduces the tendency of HbS polymerisation within the RBCs^8^. Also recent studies have shown that increased levels of HbF are associated with significant elevation in the antioxidant enzymes like superoxide dismutase (SOD), catalase, glutathione peroxidase (GPx), and reduced glutathione (GSH), with an inverse effect on lipid peroxidation, thus lowering the oxidative stress in the organs and reducing cellular injuries^9,10^. Hence, with this background an identification of genetic marker that is involved in oxidative stress pathway and which also plays a role in modulating the HbF levels will be of importance as this may assist in selecting patients with milder or severe clinical disease.

Thus in this study we have screened for the 3 polymorphisms in the *HMOX1* gene and have evaluated their association of genotypes with the HbF levels in sickle cell homozygous patients.

These 3 polymorphisms in the *HMOX1* gene rs3074372 (GT)n repeats, − 413: rs2071746 (A→T) and an intronic variation rs2071749 (A→G) have been earlier studied in disease conditions like Chronic Obstructive Pulmonary Disease (COPD), Parkinson's disease, HIV-induced CNS neuroinflammation, Cardiovascular disease, with their effect on developing a pathologic condition. Further recent study in the Brazilian population, showed that, the *HMOX1* rs2071746 genotype frequencies were 24.3% (AA), 48.6% (AT) and 27.0% (TT) in the SCA patient group and 28.2% (AA), 52.7% (AT), 19.1% (TT) in the control group. Similarly, for the (GT)n repeats, genotype frequencies of the long form repeats were higher 56.0% (LL), 37.3% (SL) as compared to the shorter repeats 6.7% (SS) in the SCA patient group. Conversely in control group, the frequencies were found to be: 53.1% (LL), 40.0% (SL) and 6.9% (SS)^11^. rs2071749 (A→G) polymorphism has not been earlier studied in SCA patients.

As there is a sparse documentation of these polymorphisms in Sickle cell anemia (SCA) patients, we selected these 3 polymorphisms, to understand their prevalence and association with HbF levels in Indian SCA patients.

## Materials and methods

### Patient selection

90 sickle cell anemia patients referred to the Department of Hematogenetics, National Institute of Immunohaematology and 50 healthy controls were enrolled in the study. The study was approved by the National Institute of Immunohaematology-Institutional Ethics Committee and the samples were obtained after a written informed consent from the patients. For patients below the age of 18 years we obtained the informed consent from parents/Legal guardians**.** All methods were carried out in accordance with relevant guidelines and regulations (declaration of helsinki). Since the HbF levels are influenced with hydroxyurea therapy, the patients on hydroxyurea therapy were excluded from this study.

### Preliminary screening

5 ml of peripheral blood EDTA samples were collected from the patients and the healthy controls. Red cell indices were measured on an automated blood cell counter (Sysmex KX-21). HbA_2_, HbF and HbS levels were measured using cation exchange HPLC on the Variant Hemoglobin Testing System (Bio-Rad Laboratories, Inc., Hercules, CA, USA).

### Molecular analysis

Genomic DNA was isolated from peripheral blood leucocytes using the QIAamp Blood Mini Kit. The HbS confirmation was carried out using Covalent Reverse Dot Blot technique^12^. 3 polymorphisms in the *HMOX1* gene based on their presumptive functional effects and on the basis of the literature survey were selected. The rs3074372 (GT)n repeats were detected using fluorescently labelled primers with primer sequence as followed:F’FAM:5′AGAGCCTGCAGCTTCT3′ and R:5′ACAAAG TCTGGCCATAGGAC 3′ and PCR was conducted^13^. The PCR products were loaded along with a size standard LIZ (− 250) (Applied Biosystems) onto ABI genetic analyser 3130 XL, under fragment analysis protocol and the fragment size was analysed by GeneMapper v4.1 (Applied Biosystems) software. The rs2071746 (A→T) polymorphism was detected by direct DNA sequencing, using BigDye terminator mix (Applied Biosystems) with already described primers and protocol^14^. The rs2071749 (A→G) polymorphism was detected by using MluCI restriction enzyme digestion (New England BioLabs)^13^.

### Statistical analysis

The genotypic and allelic frequency was calculated. Allele frequency was calculated as the number of occurrences of the test allele divided by the total number of alleles in a given group. The differences among the HbF levels within the genotypes of the each polymorphism were calculated by using non parametric test (Mann–Whitney within 2 groups) or ANOVA (more than 2 groups were involved), using GraphPad Prism version 6.0 software (GraphPad Software, Inc., San Diego, CA). The linear regression analysis was used to construct the genetic models to understand the association of the single nucleotide polymorphisms (SNPs) with the response factor (HbF levels) using SNPStats software (https://bioinfo.iconcologia.net/SNPstats)^15^. Further, SNP-SNP interaction analysis was performed by using generalised multifactor dimensionality reduction [GMDRv1.0] software, where in the most effective SNP associated with HbF level was determined.

## Results

**Figure 1:**
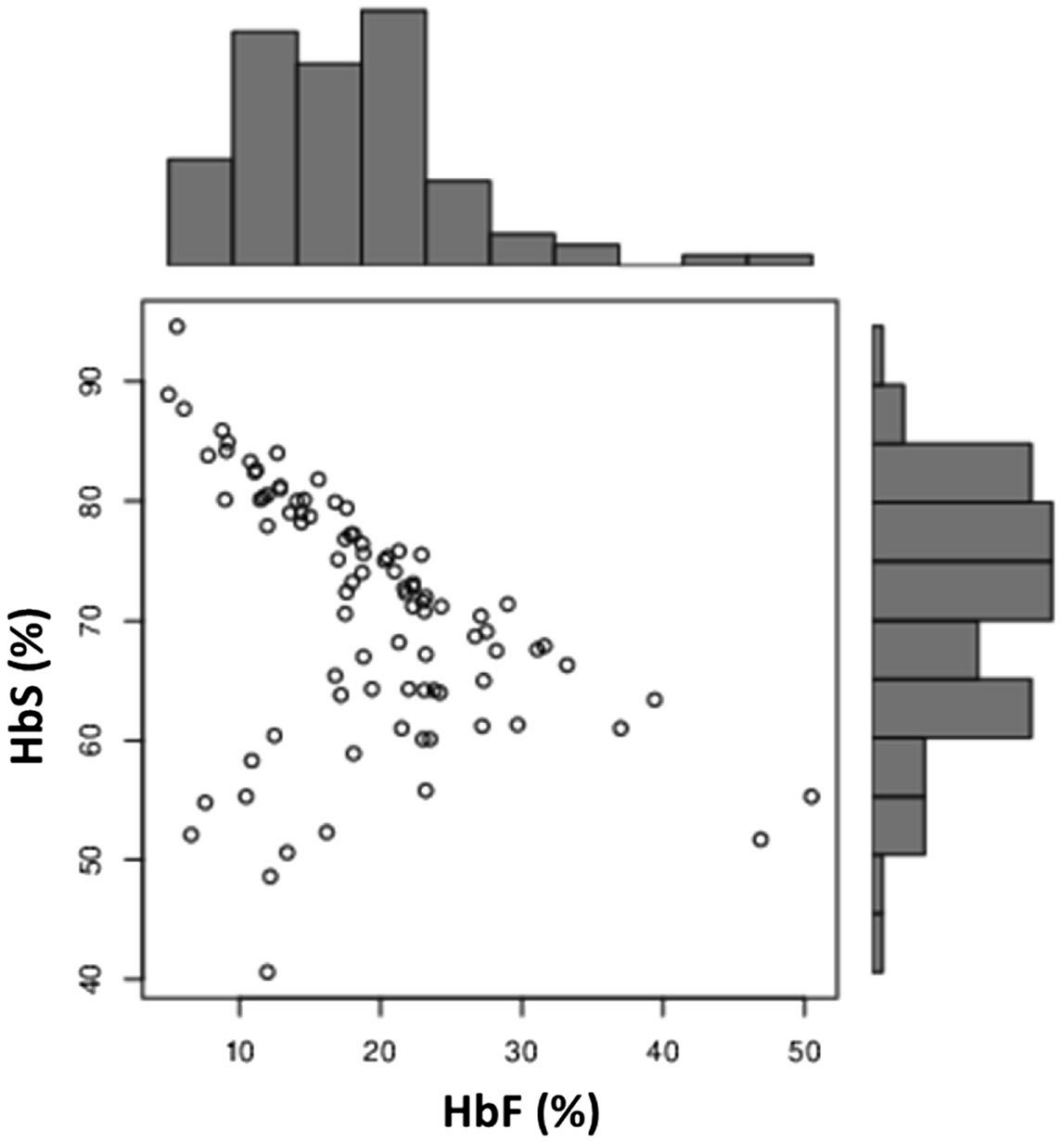
Inverse correlation between the HbF and HbS levels among the patient group. R: − 0.422, P value < 0.0001.

A total of 90 Sickle cell homozygous patients with Arab-Indian haplotype, not on hydroxyurea therapy (Age range: 1 to 41 years, mean age: 14 years) and 50 healthy controls (Age range: 5 to 52 years, mean age: 18 years) were recruited for the study. The hematological analysis was carried out. Table 1 shows the details of hematological analysis of the patient and the control group. In order to understand the effect of elevated HbF levels on the hematological indices, the patients were classified into 2 groups considering the median HbF levels of 18.1%.It was observed that the hematological indices were significantly improved in patients with higher HbF levels as compared to the other group. It was also observed that the HbS levels showed a significant negative correlation with the HbF levels [Pearson Correlation coefficient (R): − 0.422, P < 0.0001]. (Fig. 1).

**Table 1:** Hematological indices in normal and sickle cell anemia patients.

Indices	Normals (n:50)	SCA patients (n:90)	SCA patients		P value (*)
			HbF < 18.1 (n:44)	HbF > 18.1 (n:46)	
RBC (10^6^/µl)	4.6 ± 0.6	2.74 ± 0.9	2.63 ± 0.99	2.88 ± 0.99	0.23
Hemoglobin (g/dL)	13.5 ± 2.04	8.1 ± 2.4	7.6 ± 2.3	8.7 ± 2.3	**0.01**
MCV (fL)	83.9 ± 4.2	80.72 ± 10.38	78.8 ± 10.57	82.57 ± 10.2	0.12
MCH (pg)	28.9 ± 4.2	27.3 ± 4.3	26.0 ± 4.5	28.6 ± 3.6	**0.002**
MCHC(g/dl)	34.5 ± 1.5	33.13 ± 2.5	32.6 ± 2.0	33.68 ± 2.7	**0.03**
RDW (%)	13.85 ± 1.5	21.4 ± 5.6	22.4 ± 4.6	20.1 ± 5.9	**0.0008**
Platelets (10^3^/µl)	291.7 ± 38.04	306.0 ± 187 .4	352.9 ± 18.2	260.5 ± 145.1	**0.01**
HbA2 (%)	2.6 ± 0.2	2.5 ± .96	2.7 ± 0.7	2.5 ± 1.2	**0.02**
HbF (%)	0.31 ± 0.25	18.7 ± 8.4	12.23 ± 3.6	25.2 ± 6.9	** < 0.0001**
HbS (%)	–	70.5 ± 10.3	73.82 ± 12.58	67.3 ± 6.0	** < 0.0001**

*n* Number of samples in each group.

*Comparing the indices between the patients with HbF levels less and greater than 18.1%.

The genotype distribution for the three polymorphisms was consistent with the Hardy–Weinberg equilibrium. The genotype and the allelic distribution of the 3 polymorphisms [rs2071749 (A→G), rs2071746 (A→T) and rs3074372 (GT)n repeats] in both healthy and the patient group was calculated. Only the genotypic frequency of the long form of the (GT)n repeats was found to be significantly higher in the patient group [P < 0.002]. The genotypic and allelic frequencies of the long form of the (GT)n repeats were also significantly higher in SCA patients with HbF levels > 18.1 as compared to the other SCA patient group with HbF levels < 18.1% (Table 2).

**Table 2:** Genotypic and Allelic frequency determination of the 3 HMOX1 gene polymorphisms studied.

	Controls n:50 (proportion)	Sickle cell anemia			P value	Odds ratio 95% CI	Relative risk ratio 95% CI
		Overall n:90 (proportion)	HbF < 18.1% n:44 (proportion)	HbF > 18.1% n: 46 (proportion)			
**rs2071749 (A**→**G)**							
Genotypic frequencies							
AA	6 (0.12)	11 (0.12)	4 (0.09)	7 (0.15)	P*: 0.97		
AG	18 (0.36)	34 (0.38)	17 (0.38)	17 (0.37)	P^$^: 0.67		
GG	26 (0.52)	45 (0.50)	23 (0.52)	22 (0.48)			
Allelic frequencies							
A	30 (0.30)	56 (0.31)	25 (0.28)	31 (0.34)	P*: 0.89	0.94 (0.5–1.6)	0.96 (0.68–1.36)
G	70 (0.70)	124 (0.69)	63 (0.72)	61 (0.66)	P^$^: 0.52		
**rs2071746 (A**→**T)**							
Genotypic frequencies							
AA	9 (0.18)	16 (0.18)	11 (0.25)	5 (0.11)	P*: 0.82		
AT	23 (0.46)	37 (0.41)	17 (0.37)	20 (0.43)	P^$^: 0.20		
TT	18 (0.36)	37 (0.41)	16 (0.36)	21 (0.46)			
Allelic frequencies							
A	41 (0.41)	69 (0.38)	39 (0.44)	30 (0.33)	P*: 0.66	0.89 (0.5–1.5)	0.96 (0.80–1.15)
T	59 (0.59)	111 (0.62)	49 (0.56)	62 (0.67)	P^$^: 0.10		
**rs3074372 (GT)**_**n**_** repeats**							
Genotypic frequencies							
SS	24 (0.48)	19 (0.21)	13 (0.30)	6 (0.13)	P*: 0.002		
SL	19 (0.38)	42 (0.47)	20 (0.45)	22 (0.48)	P^$^: 0.11		
LL	7 (0.14)	29 (0.32)	11 (0.25)	18 (0.39)			
Allelic frequencies							
S	67 (0.67)	80 (0.44)	46 (0.52)	34 (0.37)	P*: 0.0003	0.39 (0.2–0.7)	0.72 (0.60–0.86)
L	33 (0.33)	100 (0.56)	42 (0.48)	58 (0.63)	**P**^**$**^**: 0.03**		

P*: P values calculated between the SCA and normal control samples. P^$^: P values calculated between the 2 SCA groups, those with HbF levels lower and higher than 18.1%. Bold values indicate significance at P < 0.05.

**Figure 2:**
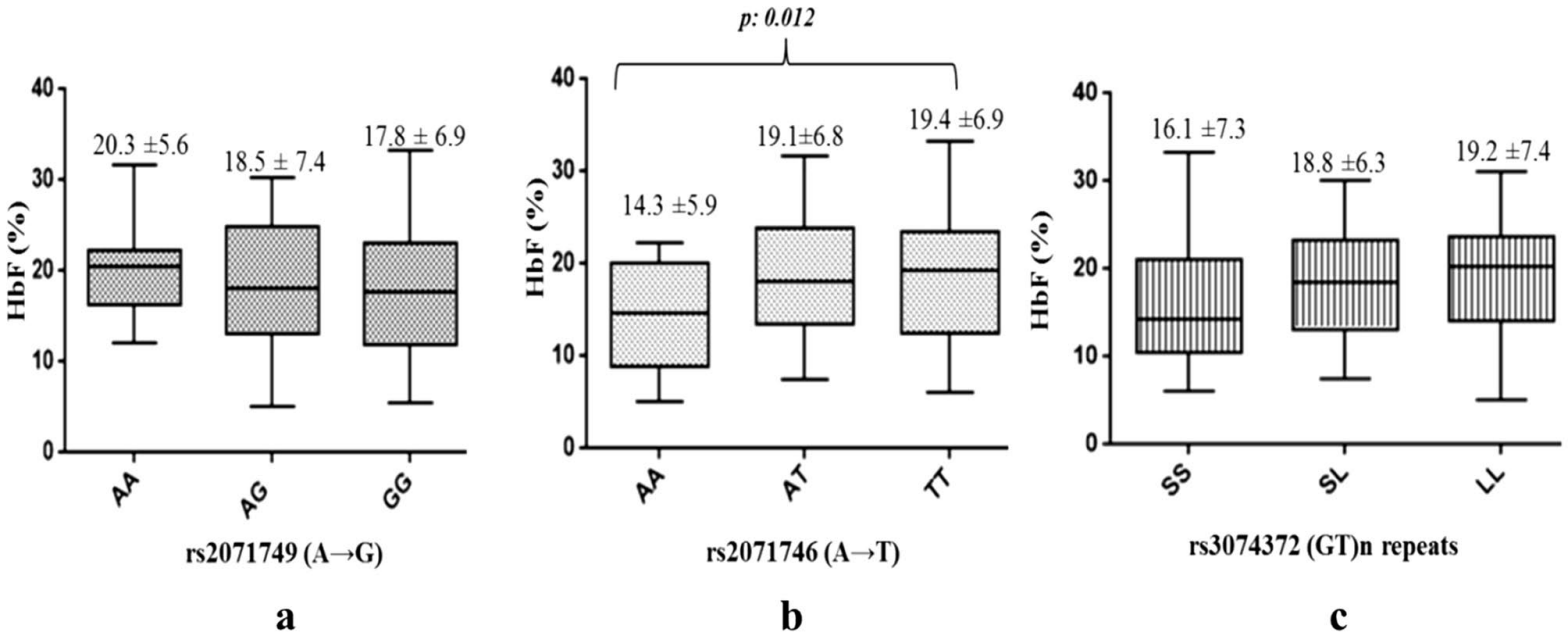
Distribution of HbF levels among genotypes of the three HMOX1 gene polymorphisms. (**a**,**c**) Represents the distribution of HbF levels among the genotypes of polymorphism rs2071749 (A→G) and rs3074372 (GT)n repeats respectively, however no statistical significance was observed. (**b**) Shows the distribution of Hb F levels among genotypes of the rs2071746:A→T, where the mutant TT genotype was found to be significantly associated with elevated HbF levels. The analysis was done using GraphPad Prism version 6.0 software (GraphPad Software, Inc., San Diego, CA).

**Figure 3:**
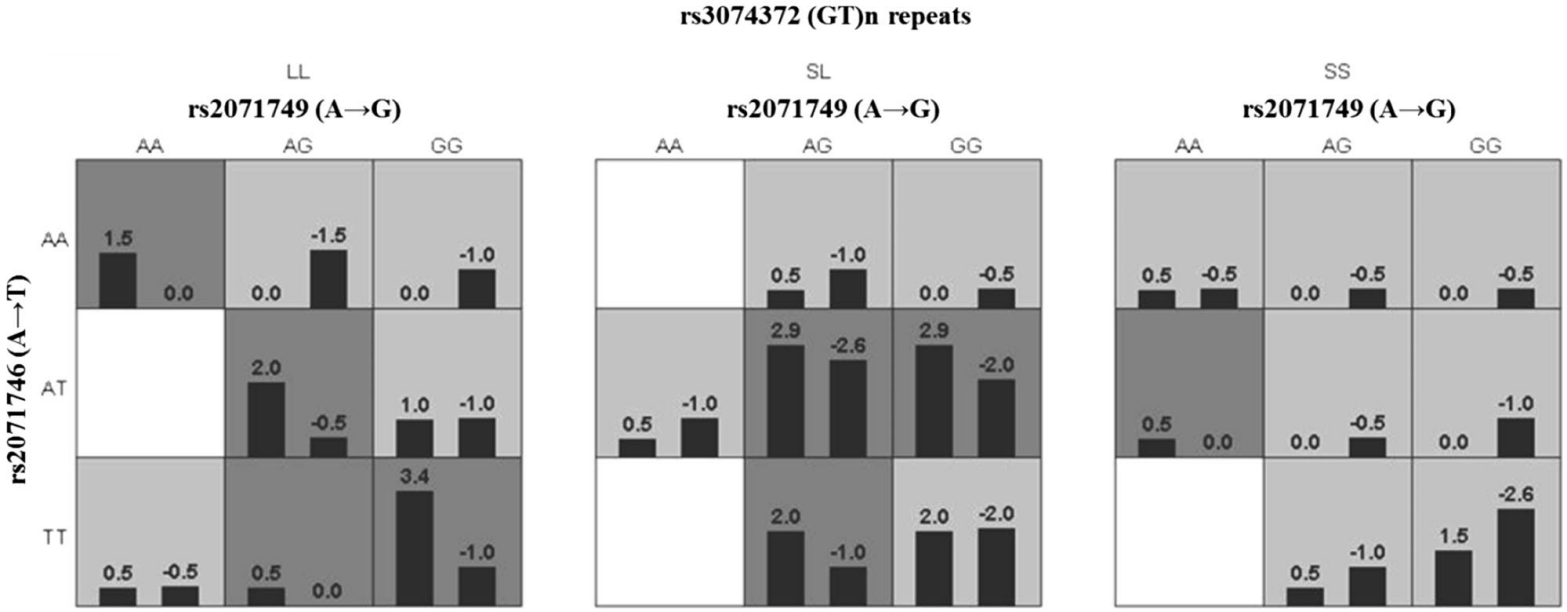
SNP-SNP interaction study by conducting generalised multifactor dimensionality reduction analysis [GMDRv1.0 software (*ibi.zju.edu.cn*)]. The left bar represents the SCA cases with higher HbF levels and the right bar represents SCA cases with lower HbF levels. The dark grey tiles represent the genotypes associated with higher HbF levels. Among the 3 SNPs , it could be observed that the presence of mutant allele T of rs2071746 (A→T) polymorphism may be associated with higher HbF levels.

The genotypic variations were then compared with the response (HbF) level in the overall patient group. The genotypes of the 2 polymorphisms: rs2071749 (A→G) and rs3074372 (GT)n repeats did not show any significant association with the HbF levels (P value: 0.64 and 0.10 respectively, using Mann Whitney non parametric test). However the mutant genotype ‘TT’ of rs2071746 polymorphism was found to be significantly associated with the HbF levels (P: 0.012, Mann Whitney non parametric test) (Fig. 2). Further, generalised multifactor dimensionality reduction [GMDR] analysis was carried out to understand the interaction of the covariates (SNPs) in elevating the HbF levels. It was observed that the presence of mutant allele T of rs2071746 A→T polymorphism was higher in SCA patients in raised HbF group (Fig. 3). Multivariate analysis using different genetic models indicated that the T allele of rs2071746 A→T variant was significant for the Co dominant and dominant genetic model with the TT genotype resulting in significant induction of HbF levels (Table 3).

**Table 3:** Association of rs2071746 with % HbF levels in SS patients using different genetic models.

Genetic model	Genotype	Response variable (s.e.) (HbF levels %)	Difference (95% CI)	P value	AIC
**Co-dominant**	TT	**20.2 (1.48)**	**0.00**	**0.048**	**597.9**
	AT	19.7 (1.38)	**− 0.74 (− 3.68 to 2.20)**		
	AA	14.2 (1.49)	**− 4.77 (− 8.58 to − 0.97)**		
**Dominant**	**TT + AT**	**20.01 (1.01)**	**0.00**	**0.015**	**596.2**
	**AA**	**14.2 (1.49)**	**− 4.41 (− 7.90 to − 0.91)**		
Recessive	TT	20.24(1.48)	0.00	0.17	600.4
	AT + AA	18.12 (1.12)	− 1.95 (− 4.71 to 0.81)		
Over dominant	TT + AA	18.43 (1.18)	0.00	0.63	602.1
	AT	19.79 (1.38)	0.68 (− 2.11 to 3.48)		

Bold values indicate significance at P < 0.05.

*SS* sickle cell anemia patients; *s.e* standard error, *CI* confidence interval; P values: obtained from logistic regression modeling after adjustment for age (Software used SNPStats), *OR* odds ratio *AIC* Akraike Information Criterion.

Thus these observations are in concordance with the univariate analysis of this SNP with the HbF levels, thus suggesting its association in elevating the HbF levels in SCA patients.

## Discussion

Sickle cell anemia is a monogenic disorder caused by the homozygosity of single β globin gene mutation at codon 6 position. The deoxygenated sickle hemoglobin has differed physiological properties and the sickle RBCs show altered rheological effects. Polymerization of HbS, precipitates in RBCs leading to disease related crises such as anemia, vaso-occlusion, acute chest syndrome, ischemia reperfusion, secondary bacterial infection and fever^16^.

Though, SCA follows a simple mendelian inheritance, the disease shows varied clinical phenotypes. This is attributed to various genetic modifiers such as coexisting α-thalassemia and the innate ability to produce elevated HbF levels^16^. Many studies have shown that there is strong association between elevated HbF levels and amelioration of the disease severity in SCA patients, as elevated HbF level delays the polymerisation of the sickle hemoglobin^17^.

Thus the effect of HbF levels on the hematological parameters and the HbS levels was first analysed. In this study, it was observed that the patients with higher HbF levels showed significantly improved hemoglobin (P: 0.01) as compared to the other group with lower HbF levels. A similar result was observed in Nigerian SCA children, where in the elevated HbF levels showed a direct relationship with hemoglobin concentration. Thus determination of HbF and its induction may be useful to maintain optimal hematological state of patients with sickle cell anemia^18^. In this study it was also observed that the sickle hemoglobin showed a significant inverse correlation with the HbF levels.

The complication of SCA involves generation of excessive free radicals, due to an electron transfer between the heme iron and oxygen, thus causing an oxidative stress^2^. The pro-oxidative and proinflammatory environment created by free heme, activates an antioxidant gene heme oxygenase 1 (*HMOX1*) to protect cellular mechanisms. Further recent a study have shown that low doses of carbon monoxide (the by product of heme metabolism) plays a key role in modulating the Nrf2 pathway that is linked to HbF regulation^19^.

Hence, we intended to analyse 3 polymorphisms located in *HMOX1* gene, and their direct association with HbF levels, as this may be of importance in predicting the disease severity of SCA patients. Study by Nacoulma et al., showed that increase in HbF levels in SCD patients, reduce the clinical manifestation of the disease by lowering the oxidative stress^20^.

The most significant SNP associated with higher HbF levels in SCA patients was found to be − 413A/T (rs2071746) polymorphism. Our study demonstrated that the mutant allele T in the heterozygous state and the homozygous form was found to be higher in both normal and SCA patients, as compared to the wild type AA genotype of − 413A/T (rs2071746) SNP. A similar frequency of the mutant allele was observed Bakr et al., in the sickle cell disease patients, where in the mutant genotype AT and TT was found to be at a higher frequency in both patients (53% and 39% respectively) and unaffected control groups (44.4% and 41.4% respectively) as compared to the wild genotype (AA) (8% and 14.1% respectively)^21^. However, the A (wild type allele) formed the major allele in the Iranian SCD patients and the normal control^22^. In the Brazilian cohort, AT genotype, was found to be the most common genotype in both patients and control group (44% and 62% respectively)^14^.

In connection to the association with the HbF levels, the mutant allele T of rs2071746 SNP, was found to be associated with elevated HbF levels in SCA patients. A similar result was observed by Gil et al., where the patients carrying the TT genotype had significantly higher levels of HbF when compared with other genotypes (P value: 0.0131). However in this contrast, in the study by Bakr et al., the TT genotype did not show any association with the HbF levels, but was related to improved clinical conditions with significant reduction in vaso-occlusive crisis (VOC), stroke, hospitalization rate , and better response to hydroxyurea treatment^21^.

The wild allele ‘A’ is reported to be associated with expression of the *HMOX1*I gene, however the mutant allele T of rs2071746 (A→T) diminishes the gene expression thus reflecting its functional significance. This polymorphism is also been well studied in the association with other diseases. In a study by Ono K et al., they observed that the AA genotype reduced the incidence of ischemic heart diseases, possibly due to high expression level of HMOX1^23^. Similarly, Ono et al*.*, have showed that AA genotype is associated with increased incidence of hypertension in women^24^.

The second polymorphism studied was the intronic variant rs2071749 (A→G). In this study, though the patients with AA genotype, showed higher HbF levels, they could not achieve significance. This polymorphism has not been studied in SCA patients, nonetheless it is found to be associated with its gene expression and the lung function. Study by Park et al., showed that A allele of this polymorphism, had a protective effect on lung function in paediatric cystic fibrosis patients^25^. In another study by Jiménez-Osorio et al., the carriers for AA genotype, were predisposed to obesity as they showed lower BMI values. The rs2071749 SNP is located within an intron of the *HMOX1* gene and, therefore, does not have an obvious functional impact^26^. However, this polymorphism is found to be in strong linkage disequilibrium with another promoter polymorphism (rs3761439) which influences the *HMOX1* gene expression, thus indirectly gaining the favourable importance^25^.

The *HMOX1* (GT)n promoter microsatellite has been evaluated for its role in a number of clinical outcomes, including cardiovascular and pulmonary diseases^27,28^. In this study, we tried to evaluate the role (GT)n promoter polymorphism with the HbF levels in SCA patients. We observed that, the longer repeats were significantly higher in SCA patients as compared to the normal control (P: 0.002). Also, the allelic frequency of longer repeats, was significantly higher in SCA patients with higher HbF levels (P: 0.03). SCA patients with longer repeats showed elevated HbF levels, however could not achieve the significance. The (GT)n repeat alleles were first studied by Bean C et al., to determine the association of this polymorphism with the clinical phenotype in SCD patients. They witnessed, that the shorter *HMOX1* (GT)*n* alleles showed a protective effect and the patients showed significant reduction in hospitalization and in acute chest syndrome^29^.

Considering the functional effect of these polymorphisms, the long form of (GT)_*n*_ repeat and the T allele of rs2071746 (A→T) SNP both located in the promoter region, have been reported to reduce the *HMOX1* gene expression leading to a decrease in the protein formation^30^. Belcher et al., highlighted the role of hemoxygenase I (HO l) in the transgenic SCD mice and showed that, the elevated or reduced expression of the HO1 enzyme may be inversely associated with hypoxia/reoxygenation–induced vascular stasis. These results suggest the encouraging role of HO in inhibiting the vascular inflammation and vaso-occlusion in SCA patients^30^.

Studies have shown that the T allele of rs2071746 (A→T) polymorphism and the long form of (GT)n repeats lead to the reduction in the *HMOX1* gene expression. Thus, a possible hypothesis for elevated HbF levels in SCA patients with T allele and long form of (GT)n repeats could be due to disrupted stress hematopoiesis of stem cells and progenitors. This leads to premature release of erythroblasts into the circulation, which in turn elevates the HbF levels^30^.Our hypothesis is supported by the review of Mabaera R et al., that the presence of a cellular stress, may activate integrated stress response, p38 mitogen-activated protein kinase (MAPK) and cyclic adenosine monophosphate (cAMP) signaling pathways which ultimately augment in indirect activation of HBG gene and increase the HbF levels^31^. Also an interlinked role of low concentration of carbon monoxide in activating the NRF2 gene transcription was described by Wang et al., as a therapeutic agent in ischemia–reperfusion brain injury^19^. NRF2 transcription factor a master regulator of cellular oxidative stress response has been shown to positively regulate the γ-globin transcription with an associated increase in HbF levels^32^. Thus, our findings, support the synergetic role of these factors in elevating the HbF levels.

HbF levels are hereditable and the phenomenon of raised HbF in SCA patients where the *HMOX1* gene polymorphism associated with HbF levels is absent, may be due to the other SNPs linked to HbF regulation like mutations within the β-globin gene cluster which includes XmnI polymorphism (− 158 C→T) or in the γ globin promoter region^33^. In Indian sickle cell anemia patients, the Arab-Indian haplotype which is linked to XmnI polymorphism, is found to be the most prevalent haplotype. However though the patients inherit the Arab Indian haplotype a wide range of HbF variability is seen. Thus, this study highlights, a presence of alternative mechanisms for HbF induction. To date, the studies have shown that increased HbF level involves 2 mechanisms. The first mechanism states that there is direct effect on the γ-globin gene expression, either by activation of the γ-globin gene transcription or by the inhibition of γ-globin gene repression. Some quantitative trait loci (QTL) have been identified as important determinants for increase in HbF levels in SCD patients such as HMIP locus on chromosome 6, the *BCL11A* locus on chromosome 2, the Xp22.2 region of the X chromosome and the 8q region on chromosome 8 and more recently *KLF1* locus^34^. Second possible mechanism is alteration of kinetics of erythroid maturation and differentiation, with release of more erythroid progenitors which predominantly have high HbF concentration^24^. With our current data, it could be speculated that *HMOX1* gene polymorphisms may promote HbF induction, through this alternative secondary mechanism independent of the γ-globin gene regulation pathway.

## Conclusions

True understanding of how the genetic variants in *HMOX1* gene functions, requires an integrative study of the expression patterns and their impact on other erythroid genes. Thus studying these SNPs will help in understanding the mechanism of HbF induction in hemoglobinopathy patients. In future the cumulative study of the genetic modifiers of fetal hemoglobin may help in forecasting of disease severity.
